# Cryopreservation of six Symbiodiniaceae genera and assessment of fatty acid profiles in response to increased salinity treatments

**DOI:** 10.1038/s41598-022-16735-w

**Published:** 2022-07-20

**Authors:** Joseph Kanyi Kihika, Susanna A. Wood, Lesley Rhodes, Kirsty F. Smith, Matthew R. Miller, Xavier Pochon, Lucy Thompson, Juliette Butler, Jessica Schattschneider, Clint Oakley, Ken G. Ryan

**Affiliations:** 1 Cawthron Institute, Private Bag 2, Nelson, 7042 New Zealand; 2 School of Biological Sciences, Victoria University of Wellington, PO Box 600, Wellington, 6140 New Zealand; 3 School of Biological Sciences, University of Auckland, Private Bag 92019, Auckland, 1142 New Zealand; 4 Institute of Marine Science, University of Auckland, Private Bag 349, Warkworth, 0941 New Zealand

**Keywords:** Biological techniques, Microbiology

## Abstract

Symbiodiniaceae are a diverse group of dinoflagellates, the majority of which are free-living and/or associated with a variety of protists and other invertebrate hosts. Maintenance of isolated cultures is labour-intensive and expensive, and cryopreservation provides an excellent avenue for their long-term storage. We aimed to cryopreserve 15 cultured isolates from six Symbiodiniaceae genera using dimethyl sulfoxide (DMSO) as the cryoprotectant agent (CPA). Under 15% DMSO, 10 isolates were successfully cryopreserved using either rapid freezing or controlled-rate freezing. Cultures that failed or had low survival, were subjected to (1) a reduction of CPA to 10%, or (2) increased salinity treatment before freezing. At 10% DMSO, three further isolates were successfully cryopreserved. At 15% DMSO there were high cell viabilities in *Symbiodinium pilosum* treated with 44 parts per thousand (ppt) and 54 ppt culture medium. An isolate of *Fugacium* sp. successfully cryopreserved after salinity treatments of 54 ppt and 64 ppt. Fatty acid (FA) analyses of *S. pilosum* after 54 ppt salinity treatment showed increased saturated FA levels, whereas *Fugacium* sp. had low poly-unsaturated FAs compared to normal salinity (34 ppt). Understanding the effects of salinity and roles of FAs in cryopreservation will help in developing protocols for these ecologically important taxa.

## Introduction

Dinoflagellate species belonging to the family Symbiodiniaceae often exist in endosymbiotic relationships with invertebrates including corals, sea anemones, jellyfish, giant clams, flatworms and benthic foraminifera^1^. Some species also exist as free-living phytoplankton in coastal environments^2^. Endosymbiotic Symbiodiniaceae are crucial for supporting the growth, survival and metabolic processes of their hosts by providing photosynthetically fixed carbon in the form of carbohydrates, sugars, starches, and amino acids^3^. Their symbiosis with stony corals is fundamental to the formation and existence of healthy coral reef ecosystems^4^. Symbiodiniaceae are genetically diverse and have historically been grouped into a number of phylogenetic lineages, i.e. clades A–J^5,6^ and numerous sub-clade types or species^7^. However, the recent taxonomic revision of the family Symbiodiniaceae has enabled the formal description of eleven genera^8^ each containing a variety of Symbiodiniaceae species with distinct ecological attributes.

Many dinoflagellate species are maintained in living culture collections. This requires continuous sub-culturing that is costly and risks strain loss, contamination, and changes in genetic integrity^9,10^. Cryopreservation, the process of preserving viable cells, tissues or microorganisms in a frozen state over extended periods of time, provides an opportunity to overcome these challenges^11^. Successful cryopreservation depends on the application of appropriate cryoprotectant agents (CPAs), freezing rates, osmotic balance and ice nucleation processes^12^. In marine microalgae, CPAs such as glycerol, dimethyl sulfoxide (DMSO) and methanol are added to avoid cellular damage due to ice formation during freezing^9,13^. The optimal CPA, equilibration time, type of freezing rates used, and thawing temperature varies between species^10,14,15^.

Successful cryopreservation of symbiotic dinoflagellates will provide a resource that can be used in research and the conservation of coral reefs^16^. Use of different cryopreservation techniques and appropriate amount of cryoprotectants have led to high cell survival of the *Breviolum* sp. ^10^. For Symbiodiniaceae species, successful cryopreservation has been reported in these genera: *Durusdinium*^17–19^, *Breviolum*^10,14,19,20^, *Gerakladium*^15,19^, *Fugacium* and *Cladocopium*^19^. However, there is a need to develop robust cryopreservation protocols for various species or sub-clades and other Symbiodiniaceae genera.

The impact of increased salinity treatment in microalgal cryopreservation is an avenue that has not been explored in sufficient detail. High salinity of the culture medium causes water to move out of the cells by osmosis facilitating dehydration during the freezing process^21^. In high salinity media, microalgae can accumulate lipids as the cells switch from active cell division to the storage of energy and these changes in production of lipids differ among species^22^. Manipulating salinity conditions prior to cryopreservation has been shown to assist in the successful freezing of some microalgal species^21^.

Many marine microalgal species can tolerate great variations of salinity, and their chemical and fatty acid (FA) composition can vary in response to such fluctuations^23^. The main roles of FAs in algae are in the maintenance of cell membrane functions, structure and metabolic processes^24^. Poly-unsaturated fatty acids (PUFAs) play important roles as structural components of membrane phospholipids^24,25^. Living organisms maintain their membranes in a semi-fluid state at the growth temperature by regulating the degree of unsaturation of the membrane lipids^26^. The plasma membrane is the primary site where considerable damage occurs as a result of chilling injury during freezing^27^. The saturated fatty acids (SFAs) help to reduce the fluidity and permeability of the cell membranes which regulates the flow of substances in and out of the cells^24^. This is due to the physical properties of these FAs compared to other FA classes^28^. However, depending on culture conditions, increasing salinity in some microalgal species outside their optimal salinity range can lead to a reduction in biomass productivity that off-sets the high lipid productivity^29^. However, changes in FA profiles amongst distinct Symbiodiniaceae genera grown in both normal and increased salinity media before cryopreservation have not been investigated.

The aim of the present study was to explore whether a method originally optimized during the cryopreservation of a *Breviolum* sp.^10^ could be applied to a range of other Symbiodiniaceae genera and whether increased salinity treatment enhances cryopreservation success in the recalcitrant cultures. It was hypothesized that: [a] Most Symbiodiniaceae cultured isolates will be successfully cryopreserved using the previously optimized protocol^10^ with DMSO as the CPA, and [b] Survival will be enhanced in selected culture isolates when grown in a high salinity medium before treatment with DMSO due to the increased dehydration of cells that results in changes in the amount and types of FA produced. Investigations of different approaches that lead to high cell viabilities during cryopreservation will help in establishing a robust freezing protocol for multiple Symbiodiniaceae species. The long-term preservation of these species will facilitate future research on their genetic capacities, life cycles, secondary metabolite production, and enhance knowledge on their endosymbiotic relationships.

## Materials and methods

### Cultivation conditions of the culture isolates

#### Normal salinity

All culture isolates from the family Symbiodiniaceae utilized in this study were provided by the Marine Symbiosis and Coral Reef Biology Laboratory at Victoria University of Wellington (New Zealand) and Cawthron Institute (CICCM, New Zealand)^30^. Twelve culture isolates were cultured in f/2 growth medium^31^ and the other three culture isolates in f/2/L1 growth medium^31,32^ as shown in Table 1. For all the initial experiments, the salinity of both f/2 and f/2/L1 growth media was maintained at normal salinity (34 ppt). All the culture isolates were grown and maintained in sterile plastic flatbottomed vessels (70 mL Labserv, Thermofisher scientific NZ) with a 12:12 light: dark cycle under 100 µmol m^−2^ s^−1^ photosynthetically active radiation light at 25 °C. All culture isolates were harvested during their late exponential phase prior to DNA extraction, cryopreservation, and FA extraction experiments.

**Table 1:** Fifteen culture isolates representing 13 distinct putative species from six genera in the family Symbiodiniaceae and their ITS-2 ribosomal RNA (rRNA) genotypes.

Culture №	Species names based on ITS-2 Identification/highest identity to known type	Growth media	ITS-2 genotype	GenBank accession number(s) of our culture isolates	GenBank accession number(s) of the closest match	Query cover/E value of the closest match in GenBank	GenBank accession number(s) of our culture isolates
				ITS-2	ITS-2		28S rDNA
1	*Durusdinium trenchii*	f/2	D1a	ON259675	KU842718	100%/5e − 138	ON263271
2	*Breviolum psygmophilum*	f/2	B2	ON259676	JN558062	100%/3e − 140	ON263272
3	*Fugacium kawagutii*	f/2	F1	ON259677	JN558068	100%/7e − 147	ON263273
4	*Breviolum psygmophilum*	f/2	B2	ON259678	JN558062	100%/3e − 140	ON263274
5	*Breviolum minutum*	f/2	B1	ON259679	ON114167	100%/2e − 142	ON263275
6	*Cladocopium* sp.	f/2/L1	C1	ON259680	MN876158	100%/4e − 139	ON263276
7	*Breviolum* sp.	f/2/L1	B1	ON259681	ON114167	100%/2e − 142	ON263277
8	*Fugacium* sp.	f/2	F5.1	ON259682	JN558065	100%/7e − 147	ON263278
9	*Effrenium voratum*	f/2	E1	ON259683	JN558086	99.65%/5e − 143	ON263279
10	*Fugacium* sp.	f/2	F5.2	ON259684	AM748594	97%/1e − 143	ON263280
11	*Symbiodinium microadriaticum*	f/2	A1	ON259685	MH211592	100%/1e − 128	ON263281
12	*Symbiodinium necroappetens*	f/2	A13	ON259686	KT820174	100%/1e − 128	ON263282
13	*Symbiodinium tridacnidorum*	f/2	A6	ON259687	EU449036	100%/2e − 126	ON263283
14	*Symbiodinium pilosum*	f/2	A2	ON259688	AF333506	100%/1e − 129	ON263284
15	*Symbiodinium tridacnidorum*	f/2/L1	A3	ON259689	JN558093	100%/2e − 126	ON263285

The culture isolates were grown in both f/2 and f/2/L1 media and the GenBank accession numbers of both ITS-2 and 28S rRNA gene sequences are provided. The GenBank accession numbers of the species that is the closest match and its query cover/E value in Nucleotide BLAST are provided.

#### High salinity treatments

From our study cultures, five isolates from the genus *Symbiodinium*; *S. microadriaticum* [ITS-2 type A1], *S*. *pilosum* [A2]*, S*. *tridacnidorum* [A3], *S*. *tridacnidorum* [A6] and *S*. *necroappetens* [A13] and one isolate from the genus *Fugacium* (*Fugacium* sp. [F5.2]), were subjected to higher salinity treatments. All of these *Symbiodinium* isolates had failed to survive after thawing during the initial cryopreservation experiments using 15% DMSO while *Fugacium* sp. [F5.2] had very low cell viability after thawing. The salinity of the f/2 and f/2/L1 media was increased by adding appropriate amounts of NaCl (Emsure, Denmark) to reach either 44 parts per thousand (ppt), 54 ppt or 64 ppt as described by van der Merwe et al.^33^. These culture isolates were then harvested during their late exponential phase for cryopreservation experiments.

### DNA extraction, Polymerase chain reaction and DNA sequencing

Due to the complex diversity of the family Symbiodiniaceae, a phylogenetic analysis of the nuclear ribosomal large subunit (28S) rRNA and the nuclear ribosomal Internal Transcribed Spacer region 2 (ITS-2) rRNA was undertaken to identify all cultures isolates investigated. Molecular analyses (DNA extraction, Polymerase Chain Reaction [PCR] set-up, template addition and PCR amplification) were conducted under sterile conditions with sequential workflow to ensure there was no contamination. DNA extraction, PCR set-up and template addition was carried out in separate rooms equipped with ultra-violet sterilisation.

For each Symbiodiniaceae strain, a subsample (20 mL) was taken from a densely grown culture and transferred to a 50 mL centrifuge tube (Corning CentriStar, China) and centrifuged (3000 × *g*, 10 min). The supernatant was carefully discarded, and 1 mL of the dense culture isolate was transferred into a sterile 1.7 mL microtube (Axygen, Wujiang, China). The concentrated culture isolate was then centrifuged (3000 × *g*, 10 min) and all the supernatant discarded. The isolated pellet was stored in − 20 °C before DNA extraction. The pellet was lysed manually using a bead beater machine (1600 MiniG Spex SamplePrep, New Jersey, United States) and DNA was extracted using the DNeasy PowerSoil Kit (QIAGEN, Hilden, Germany) following the protocol from the manufacturer.

Two markers were selected for PCR and sequencing analyses: the 28S rRNA gene using forward LSU1F primer 5′-GCG GAG GAA AAG RAA CTA A-3′^34^ and reverse LO primer 5′-GCT ATC CTG AGR GAA ACT TCG-3′^35^ and the ITS-2 rRNA region using forward primer 5′-GTG AAT TGC AGA ACT CCG TG-3′^36^ and ITS-2-rev2 reverse primer 5′-GCC TCC GCT TAC TTA TAT GCT T-3′^37^. Polymerase chain reactions were performed in 50 µL reaction volume for each sample with the reaction mixture containing 25 µL of 2× PCR MyFi Mix (Bioline, London, UK), 21 µL of nuclease free water (AM9937; Ambion, CA, USA), 1 µL (10 µM) of each primer, 1 µL of bovine serum albumin (BSA, 20 mg mL^−1^, Sigma-Aldrich, Auckland, New Zealand) and 1 µL of template DNA. The PCR cycling conditions were held at 94 °C for 3 min, followed by 35 cycles of 94 °C for 20 s, 52 °C for 20 s, 72 °C for 30 s and finally, an extension step of 5 min at 72 °C. During the PCR reaction, negative PCR controls containing nuclease free water as a template were run alongside the samples. All the amplified products were then purified using NucleoSpin Gel and PCR clean up kits (Macherey–Nagel, Düren, Germany) and sent to the Genetics Analysis Services (Dunedin, New Zealand) for direct bi-directional Sanger sequencing.

### Phylogenetic analyses of the culture isolates

Forward and reverse sequences were inspected and aligned to generate consensus sequences using Geneious Prime v11.0.11 (Biomatters, Auckland, New Zealand). Consensus 28S rDNA sequences (n = 15) were manually aligned to an existing alignment dataset^38^ before performing a ClustalW alignment on the final dataset using BioEdit v7.2.0.^39^. Three additional 28S sequences from the recently described Symbiodiniaceae clade J^6^, were also incorporated into the alignment. To estimate the best-fit model of evolution and Maximum-likelihood (ML), all the analyses were performed with the use of Mega X v10.1.8^40^. The initial trees for the heuristic search were obtained automatically by applying both Neighbor-Join and BioNJ algorithms to a matrix of pairwise distance estimated using Tamura-Nei model, and then selecting the topology with superior log likelihood value. A discrete Gamma distribution was used to model evolutionary rate differences among sites (5 categories; + G parameter = 0.3631). The reliability of internal branches was assessed using 100 bootstraps method^41^.

Consensus ITS-2 sequences (n = 15) enabled identification of Symbiodiniaceae culture isolates at finer taxonomic level (type or species) using BLASTn in GenBank. Accession numbers of all sequences produced in this study are shown in Table 1.

### Cryoprotectant agent

The CPA used during freezing experiments was DMSO (≥ 99.9% pure, Sigma-Aldrich, France). CPA solutions were prepared at double the target concentration in sterilized appropriate growth medium for each culture and added to the cultures in a 1:1 ratio to give final concentrations of 10% and 15% (v/v) of the CPA.

### Cryopreservation experiments

Two different concentrations of DMSO and two freezing methods were used for 15 cultured isolates from the family Symbiodiniaceae. The cultures that did not cryopreserve or had a low post-thaw viability were subsequently grown in media with increased salinity to explore its effect during their cryopreservation. The FA profiles of all the culture isolates grown in normal salinity and the isolates that had high cell viabilities post-thaw after being treated with high salinity were analysed and compared to explore whether increased salinity contributes to cryopreservation success.

Three experiments were done: Part A: Cryopreservation experiments with a final concentration of 15% DMSO with normal salinity culture isolates, Part B: Cryopreservation experiments using a final concentration of 10% DMSO for the culture isolates that failed to survive and had low cell viabilities post-thawing (< 15%) in part A and lastly Part C: Cryopreservation experiments using a final concentration of 15% DMSO and high salinity treated culture isolates that failed to survive and had low cell viabilities post-thawing (< 15%) in part A.

During these experiments, aliquots (1 mL) of each culture in their late exponential phase were pipetted into separate 5 mL sterile glass test tubes in triplicates in a laminar flow cabinet. For each culture, 100 µL aliquots of appropriately diluted at double the desired final concentration of DMSO was added to the tubes every 1 min with gentle agitation after each addition up to the 10th min (RT, 30 µmol m^−2^ s^−1^ PAR) until a final 1:1 dilution of culture medium to CPA was obtained^10^. After the last addition, the glass tubes were stoppered and incubated in the dark (30 min). These cultures were aspirated into cryopreservation straws (0.5 cc, IMV, France), plugged with coloured polyvinyl chloride powder, and placed in water (20 °C) to set the powder. The straws were then wiped dry before the freezing procedure.

Two main cryopreservation methods were applied as detailed in Kihika et al. ^10^.

- Rapid freezing method: The straws containing the DMSO treated cultures isolates were arranged horizontally on a metal rack fitted onto polystyrene floats measuring (41 × 14 × 4 cm, l × w × h)^10^. Each rack was placed over the liquid nitrogen bath (45 × 30 × 6 cm, l × w × h) for 10 min before the straws were plunged into liquid nitrogen^10^.
- Controlled-rate freezing method: The straws were placed in a controlled-rate freezer (Cryologic Pty, Mt Waverley, Australia) which cooled the culture isolates from 20 to − 40 °C at a rate of 1 °C min^−1^. The straws were maintained at − 40 °C for 10 min before plunging into liquid nitrogen^10^.

In both cryopreservation methods, the cooling rate difference was 10 min for rapid freezing and 1 h 30 min for the controlled rate freezer. All the frozen straws were stored in a dewar containing liquid nitrogen for one week.

### Thawing and culture isolates recovery

After storage, triplicate straws of each culture isolate were retrieved and thawed by rapidly transferring into a water bath (20 °C) for 2 min until all visible ice melted. A tissue moistened with 70% ethanol was used to wipe the straws dry^10^. The straw contents were transferred to a sterile plastic flatbottomed vessel and individually diluted by stepwise addition of 500 µL of their growth medium at normal salinity (34 ppt), each minute for 10 min (total volume 5 mL)^10^. The culture isolates that had been treated with higher salinities were grown in normal salinity growth media after thawing. All culture isolates were incubated in the dark (30 min) to equilibrate before adding a final volume of 5 mL of their respective growth medium. The culture isolates were transferred to dark conditions at ⁓ 20 °C for 24 h, followed by a further 48 h under 27 µmol m^−2^ s^−1^ red light (OSRAM L18W/60, Germany) to facilitate the recovery of the dinoflagellate cells^10^. Finally, growth media (~ 40 mL) was added to the recovering culture isolates, which were then incubated in their normal standard light growing conditions.

### Cell viability assessment tests

A viability test was conducted for each cultured strain after the recovery phase on the third day to determine cell survival after freezing. The cells were carefully resuspended in the plastic flatbottomed vessel before cell counting was done^10^. A subsample (1 mL) of the cell culture was taken and an appropriate serial dilution was made following the method used by Kihika et al. ^10^. Lugol’s iodine (10 µL) was added to the diluted culture to fix the living cells for easier counting under a microscope. The diluted subsample (100 µL) was placed on a glass slide in triplicate and the fixed cells allowed to settle for 30 min^10^. An inverted microscope was used to enumerate viable fixed cells (stained dark brown), and dead cells (colorless)^10^.

### Fatty acids (FAs) extraction and identification

The FAs were extracted and analysed for all the culture isolates grown in normal salinity. A further two culture isolates grown at specific high salinities were also tested; *S*. *pilosum* [A2] was grown in two different salinity treatments of both 44 ppt and 54 ppt, and *Fugacium* sp. [F5.2] was grown in 54 ppt salinity. These culture isolates and treatments were selected for FA analysis because cells had successfully cryopreserved with viabilities of (> 50%). No culture isolate growing at 64 ppt salinity was selected for FA analysis due to low viability or lack of survival post-thawing. All the FAs were extracted from these culture isolates before cryopreservation experiments. All the culture isolates were grown in Erlenmeyer flask (500 mL) before harvesting for FAs extraction. The culture isolates were then transferred to 50 mL centrifuge tubes (Corning CentriStar, China) and centrifuged (3000 × *g*, 10 min). The supernatant was then discarded, and the dense culture isolate was resuspended and transferred into a 1.7 mL microtubes (Axygen, Mexico). The culture isolates were centrifuged (3000 × *g*, 10 min) and the supernatant discarded. The final isolated pellets were weighed from (60 to 120 mg) and stored in − 20 °C before FAs extraction.

The pellet samples (wet weight 60–120 mg) were directly added to 3 mL of methylating solution (methanol:dichloromethane:concentrated hydrochloric acid 10:1:1 v/v/v) made within 7 days. The tube was capped under Teflon, vortexed, and placed in a heating block (100 °C, 2 h). When the solution had cooled, 1 mL of Milli-Q water was added. The solution was extracted with 2 mL of hexane:dichloromethane (4:1 v/v) by shaking and then vortexing, followed by centrifugation (2000 × *g*, 5 min). The fatty acid methyl esters (FAME) occupying the upper organic layer was blown down and transferred to a vial. The 2 mL extractions were repeated twice^42^. After adding internal standard solution (19:0 FAME)^42^, samples were injected from this solution into a gas chromatograph (GC, Agilent Technologies Australia, Victoria, Australia).

FAME samples were run in accordance with AOAC official methods 963.22 “Methyl Esters of fatty acids in oils and fats”^43^. In brief, FAME was analysed by gas chromatography (GC, Agilent Technologies, Victoria, Australia) performed using an Agilent 6890 with an Agilent SP-2560 silica capillary column (DKSH New Zealand Limited, Auckland, New Zealand) (100 m × 0.25 mm i.d., 0.2 µm film thickness) and peak area determined by flame ionized detection (FID). The samples each (1 µL) were then injected via a split injector at 260 °C. The column temperature program was: 220 °C at 17 min, then raised by 2.8 °C min^−1^ to 240 °C and held for 5 min. FAs were linked to an external commercial FA standard (Supelco 37 Component FAME Mix, Merck, Auckland, NZ) with the use of ChemStation software (Version A10.02, Agilent, Auckland, NZ). Nitrogen was the carrier gas. To determine the FAs composition, the following formula with minor modifications was used: C_fa_ = (A_fa_/A_is_) × (C_is_/V_s_)/RRF, where C_fa_ was the amounts of individual FAs, A_fa_ is the chromatographic area units of the FA being investigated. A_is_ is the chromatographic area units for internal standard, C_is_ is the concentration of the internal standard used in µg/mL and V_s_ is the volume in mL of the Symbiodiniaceae sample used. The relative response factor (RRF) for each peak was determined from a commercial, equal-weight standard^44^. The mass of FAME was represented as mg 100 g^−1^ wet weight.

### Descriptive analyses

All of our study data were analysed using R software (4.0.3). To determine if there was a significant difference between the cell viabilities of the culture isolates from the two freezing techniques, unpaired t-tests were undertaken (p < 0.05) and a boxplot used to show the median distribution of the cell survival after freezing. P-values of less than 0.05 was considered to be statistically significant. For the FAs, only descriptive analyses were done. All differences in the production of mean FA classes between normal and increased salinity treatments were represented in bar graphs. Due to the large number of culture isolates and the high cost of FA analysis, there was no replication in this part of the study and only descriptive analysis was possible. All FAs from individual samples were pooled by FA class and compared by salinity (Fig. 4). The changes in FA profiles in the high salinity culture isolates when compared to normal salinity were displayed in bar graphs and the Hierarchical clustering of the FA profiles was undertaken using Bray–Curtis similarity and results presented as a CLUSTER dendrogram. The number of cluster groups (k-means) for the dendrogram was selected using both the elbow method and the gap statistic^45,46^. SIMPER analysis on PAST software (4.03) was used to identify the species that best explained the differences between the FA classes, and the dominant FA profiles.

## Results

### Phylogenetic characterisation of Symbiodiniaceae cultures

**Figure 1:**
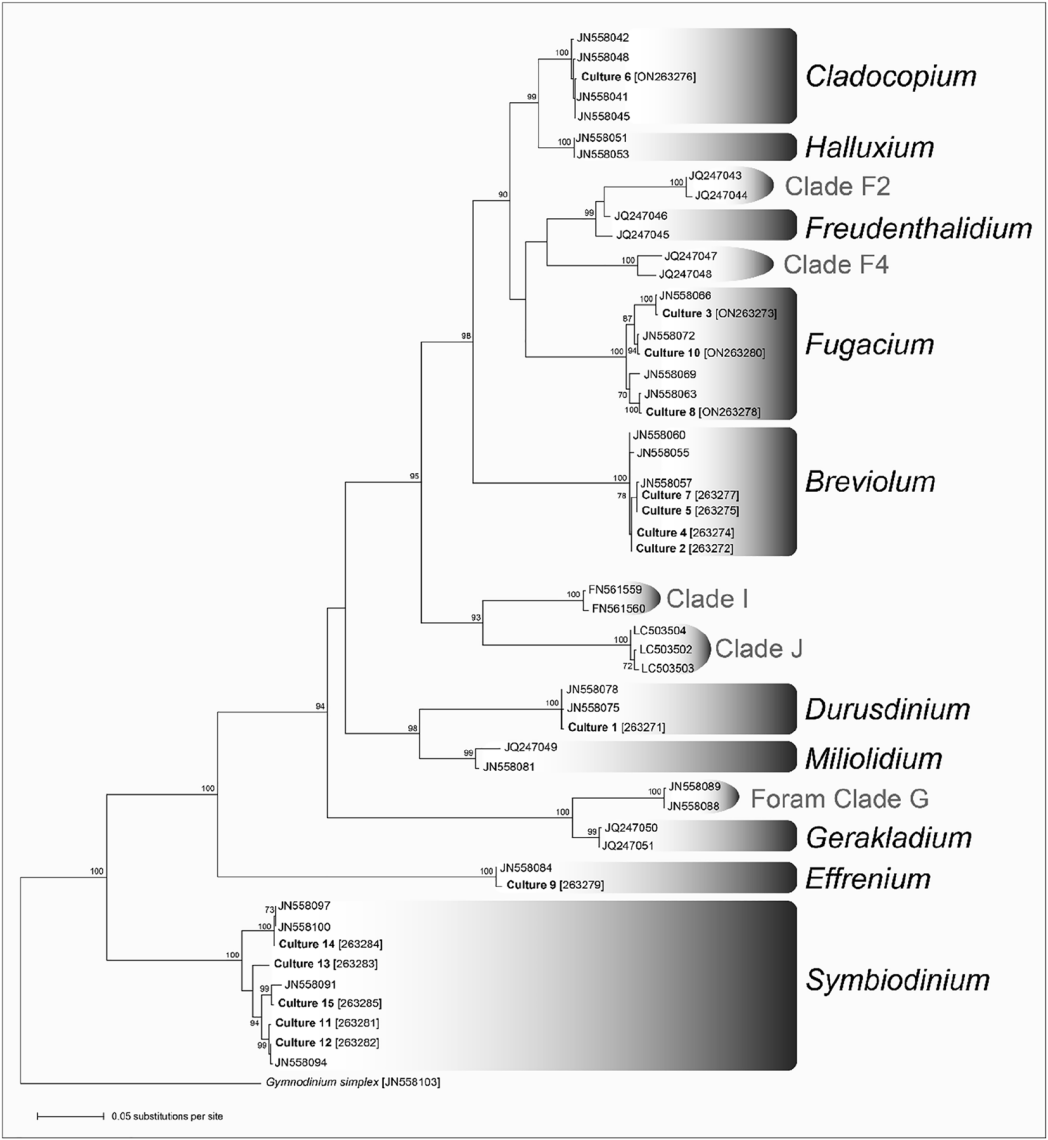
Best Maximum likelihood (ML) topology for the family Symbiodiniaceae based on nuclear large subunit ribosomal 28S rRNA gene, showing the phylogenetic placement of the 15 putative culture isolates (Cultures 1–15 in bold) together with their GenBank accession numbers as provided in Table 1 among the Symbiodiniaceae genera. Numbers at nodes represent the ML bootstrap pseudoreplicate (BP) values, excluding BP values lower than 70%. The phylogram was rooted using the dinoflagellate *Gymnodinium simplex*.

Phylogenetic analysis of the 721 base-pair 28S rRNA gene fragment showed that the fifteen cultured isolates belonged to six Symbiodiniaceae genera (Fig. 1) including *Symbiodinium* (n = 5), *Breviolum* (n = 4), *Fugacium* (n = 3), *Effrenium* (n = 1), *Durusdinium* (n = 1) and *Cladocopium* (n = 1). Finer scale identification using the ITS-2 marker indicated that the cultures belonged to 13 putative distinct Symbiodiniaceae species (Table 1).

### Cryopreservation results

#### Part A: Freezing experiments using 15% DMSO

##### Rapid freezing technique

Nine Symbiodiniaceae culture isolates were successfully cryopreserved using the rapid freezing method (Table 2). *Breviolum psygmophilum* [B2] had the best survival rate post-thawing (cell viability 87.8 ± 4.0%), followed by *E. voratum* [E1] (74.0 ± 7.8%). Low cell viabilities were observed in *D. trenchii* [D1a] and *Breviolum* sp. [B1] (16.0 ± 1.3% and 10.3 ± 1.7% respectively; Table 2). After thawing, no viable cells were observed for the six remaining culture isolates (Table 2).

**Table 2:** Cell viabilities after the rapid freezing and controlled-rate freezing techniques using 15% dimethyl sulfoxide (DMSO) as the cryoprotectant agent (CPA).

Species names	ITS-2 genotype	Post-thaw cell viabilities (% ± SD)	
		Rapid freezing	Controlled- rate freezing
		15% DMSO	15% DMSO
*Durusdinium trenchii*	D1a	16.0 ± 1.3	52.7 ± 4.8
*Breviolum psygmophilum*	B2	87.8 ± 4.0	34.0 ± 3.3
*Fugacium kawagutii*	F1	57.3 ± 6.1	50.3 ± 4.1
*B. psygmophilum*	B2	45.7 ± 6.6	62.1 ± 6.5
*B. minutum*	B1	53.6 ± 2.2	15.6 ± 0.4
*Cladocopium* sp.	C1	61.3 ± 5.6	26.7 ± 3.5
*Breviolum* sp.	B1	10.3 ± 1.7	41.2 ± 4.3
*Fugacium* sp.	F5.1	43.8 ± 6.3	NVC
*Effrenium voratum*	E1	74.0 ± 7.8	44.4 ± 2.1
*Fugacium* sp.	F5.2	NVC	15.0 ± 0.9
*Symbiodinium microadriaticum*	A1	NVC	NVC
*S. necroappetens*	A13	NVC	NVC
*S. tridacnidorum*	A6	NVC	NVC
*S. pilosum*	A2	NVC	NVC
*S. tridacnidorum*	A3	NVC	NVC

Values are mean percentage of three replicates (n = 3) ± standard deviation (SD).

*NVC* no viable cells observed.

##### Controlled-rate freezing technique

Nine Symbiodiniaceae culture isolates were successfully cryopreserved using the controlled-rate freezer method (Table 2). One isolate of *B*. *psygmophilum* [B2] had the best survival rate after thawing (62.1 ± 6.5%). *Breviolum minutum* [B1] and *Fugacium* sp. [F5.2] recorded very low cell viabilities (15.6 ± 0.4% and 15.0 ± 0.9% respectively; Table 2). After thawing, no viable cells were observed from the six remaining culture isolates (Table 2).

#### Part B: Freezing experiments using 10% DMSO

Five Symbiodiniaceae culture isolates from the genus *Symbiodinium* that failed to cryopreserve at 15% DMSO with both freezing methods and one culture from genus *Fugacium* (*Fugacium* sp. [F5.2]) that only cryopreserved using the controlled-rate freezer with a low cell viability with 15% DMSO were all cryopreserved with 10% DMSO.

##### Rapid freezing technique

One culture isolate from the genus *Fugacium* and three culture isolates from the genus *Symbiodinium* were successfully cryopreserved (Table 3). *Fugacium* sp. [F5.2] had the best survival rate (76.0 ± 4.7%), followed by *S*. *necroappetens* [A13], *S*. *microadriaticum* [A1] and *S*. *pilosum* [A2] survival rates of 62.2 ± 4.7, 25.0 ± 1.4 and 23.1 ± 3.9, respectively (Table 3).

**Table 3:** Cell viabilities after the rapid freezing and controlled-rate freezing techniques using 10% dimethyl sulfoxide (DMSO) as the cryoprotectant agent (CPA).

Species names	ITS-2 Type	Post-thaw cell viabilities (% ± SD)	
		Rapid freezing	Controlled- rate freezing
		10% DMSO	10% DMSO
*Fugacium* sp.	F5.2	76.0 ± 4.7	65.1 ± 6.3
*Symbiodinium microadriaticum*	A1	25.0 ± 1.4	NVC
*S. pilosum*	A2	23.1 ± 3.9	NVC
*S. tridacnidorum*	A3	NVC	NVC
*S. tridacnidorum*	A6	NVC	NVC
*S. necroappetens*	A13	62.2 ± 4.7	NVC

Values are mean percentage of three replicates (n = 3) ± standard deviation (SD) of the mean.

*NVC* No viable cells observed.

##### Controlled-rate freezing technique

*Fugacium* sp. [F5.2] was successfully cryopreserved with a survival rate of 65.1 ± 6.3% (Table 3).

#### Part C: Freezing experiments using 15% DMSO and high salinity treatment

##### Rapid freezing technique

***Symbiodiniaceae cultures grown in 44 ppt salinity media***

Only *S*. *pilosum* [A2] was successfully cryopreserved with a post-thaw viability (64.9 ± 5.3%; Table 4).

**Table 4:** Cell viabilities after rapid freezing with salinity treated culture isolates using 15% dimethyl sulfoxide (DMSO) as the cryoprotectant agent (CPA).

Species names	ITS-2 type	Post thaw viabilities after Salinity treatment		
		44 ppt	54 ppt	64 ppt
*Fugacium* sp.	F5.2	NVC	57.4 ± 2.7	22.5 ± 4.9
*Symbiodinium microadriaticum*	A1	NVC	NVC	NVC
*S. pilosum*	A2	64.9 ± 5.3	75.5 ± 7.2	NVC
*S. tridacnidorum*	A3	NVC	NVC	NVC
*S. tridacnidorum*	A6	NVC	NVC	NVC
*S. necroappetens*	A13	NVC	NVC	NVC

Values are mean percentage of three replicates (n = 3) ± standard deviation (SD) of the mean.

*NVC* no viable cells observed.

***Symbiodiniaceae cultures grown in 54 ppt salinity treatment***

*Symbiodinium pilosum* [A2] and *Fugacium* sp. [F5.2] were successfully cryopreserved with high post-thaw viabilities (75.5 ± 7.2% and 57.4 ± 2.7%, respectively; Table 4).

***Symbiodiniaceae cultures grown in 64 ppt salinity treatment***

Only *Fugacium* sp. [F5.2] was successfully cryopreserved with a post-thaw cell viability of 22.5 ± 4.9% (Table 4).

##### Controlled-rate freezer method

***Symbiodiniaceae cultures grown in 44 ppt salinity media***

*Symbiodinium pilosum* [A2] and *Fugacium* sp. [F5.2] were successfully cryopreserved (37.8 ± 2.8% and 33.1 ± 8.2% respectively; Table 5).

**Table 5:** Cell viabilities after controlled-rate freezing with salinity treated culture isolates using 15% Dimethyl sulfoxide (DMSO) as the cryoprotectant agent (CPA).

Species names	ITS-2 Type	Post thaw viabilities after Salinity treatment		
		44 ppt	54 ppt	64 ppt
*Fugacium* sp.	F5.2	33.1 ± 8.2	NVC	NVC
*Symbiodinium microadriaticum*	A1	NVC	NVC	NVC
*S. pilosum*	A2	37.8 ± 2.8	44.1 ± 6.9	NVC
*S. tridacnidorum*	A3	NVC	NVC	NVC
*S. tridacnidorum*	A6	NVC	NVC	NVC
*S. necroappetens*	A13	NVC	NVC	NVC

Values are mean percentage of three replicates (n = 3) ± standard deviation (SD) of the mean.

*NVC* no viable cells observed.

***Symbiodiniaceae cultures grown in 54 ppt salinity treatment***

Only *S*. *pilosum* [A2] was successfully cryopreserved (44.1 ± 6.9%; Table 5).

***Symbiodiniaceae cultures grown in 64 ppt salinity treatment***

When the selected Symbiodiniaceae culture isolates were grown in f/2 media set at 64 ppt salinity, none of the treated strains survived (Table 5).

### Cell viabilities between rapid freezing and controlled-rate freezing

The cell survival of all the successfully cryopreserved Symbiodiniaceae culture isolates grown in normal salinity media after both rapid freezing and the controlled-rate freezing techniques was investigated. The cell viabilities of the culture isolates cryopreserved with rapid-freezing were significantly higher when compared to those cryopreserved with the controlled-rate freezer (unpaired t-test, p < 0.05; cell survival after rapid freezing: 50.0 ± 24.4%, cell survival after controlled-rate freezing: 38.0 ± 16.1%).

### Fatty acid (FA) profiles

Three classes of FAs were observed: saturated fatty acids (SFAs), monounsaturated fatty acids (MUFAs) and polyunsaturated fatty acids (PUFAs). Full FA profiles are given in Supplementary Information excel Table S1. A cluster dendrogram (based on their FA profiles) was produced for all the culture isolates grown in normal salinity and for the two culture isolates *Fugacium* sp. [F5.2] and *S*. *pilosum* [A2] that survived cryopreservation after increased salinity treatments.

**Figure 2:**
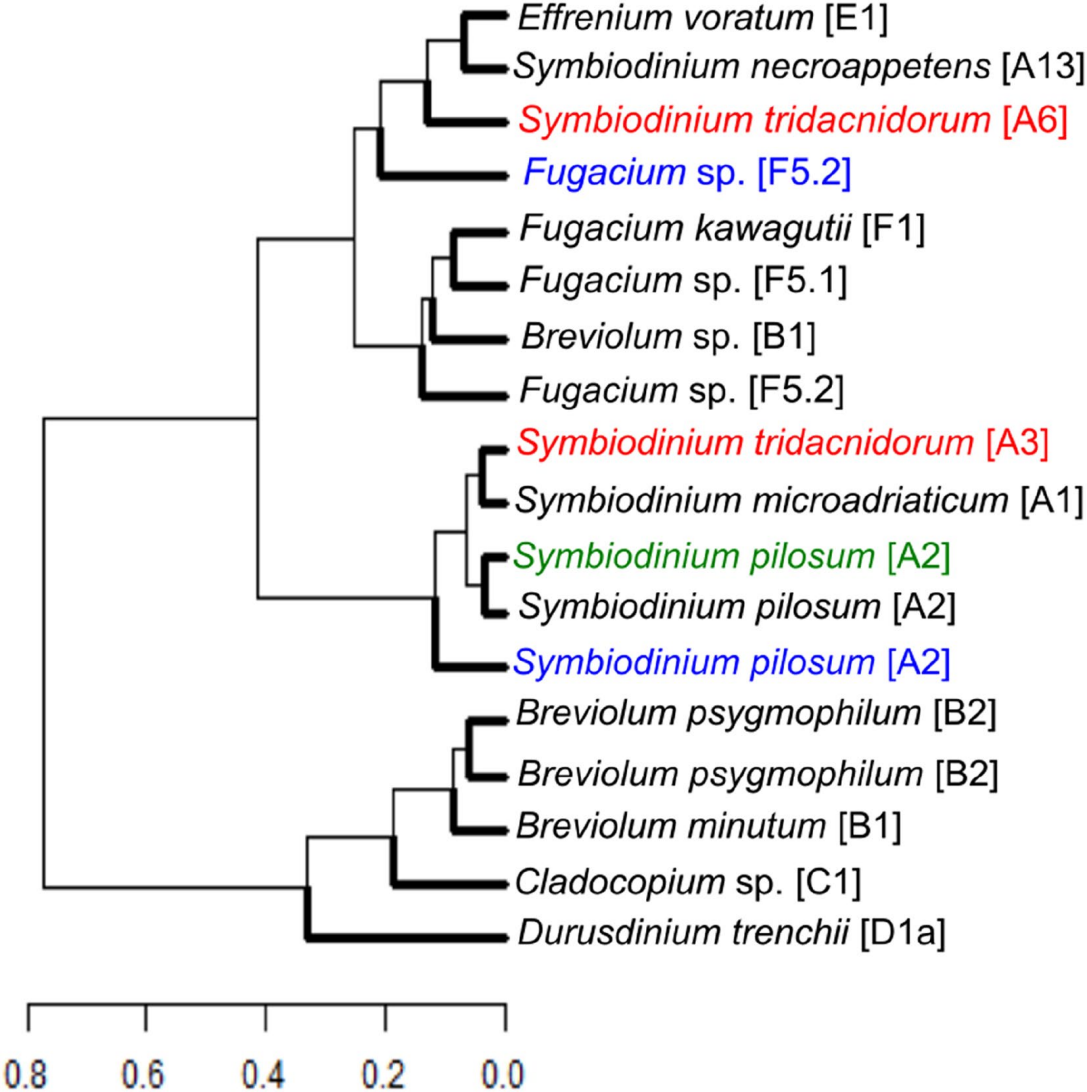
Cluster analysis dendrogram based on fatty acid (FA) profiles using the hierarchical relationship among 15 culture isolates grown at normal salinity (black font) and three selected culture isolates grown at high salinities. The six different colours of the dendrogram branches represents the six clusters for the FAs similarities among the culture isolates. Culture isolates in blue font = *Fugacium* sp. [F5.2] and *Symbiodinium pilosum* [A2] were grown in 54 ppt salinity respectively. The culture isolate in green font = *S. pilosum* [A2] was grown in 44 ppt salinity. The two culture isolates in red font = *S. tridacnidorum* [A6] and *S. tridacnidorum* [A3] did not survive freezing while all the other culture isolates successfully cryopreserved.

Two major clusters were observed that were further grouped into six subclusters that were calculated using a combination of the elbow method and gap statistic (Supplementary Information Fig. S1). The *S*. *pilosum* [A2] culture isolates grown in normal salinity and in 44 ppt salinity expressed similar FA profiles that were different to the culture isolate grown in 54 ppt salinity. For *Fugacium* sp. [F5.2], increased salinity treatment of 54 ppt resulted in the culture expressing different FA profiles that placed the isolate in a different cluster from the other cultures in the same genus *Fugacium* (Fig. 2). Three cultures of genus *Breviolum* were found in a similar FA cluster apart from *Breviolum* sp. [B1], which expressed a similar FA to that of the genus *Fugacium* (Fig. 2). *Symbiodinium tridacnidorum* [A6] and *S*. *necroappetens* [A13] were found in a FA cluster that was different from other five culture isolates in the genus *Symbiodinium*. *Durusdinium trenchii* did not cluster with any of the other culture isolates (Fig. 2).

### Fatty acid composition in all culture isolates at normal and high salinities

**Figure 3:**
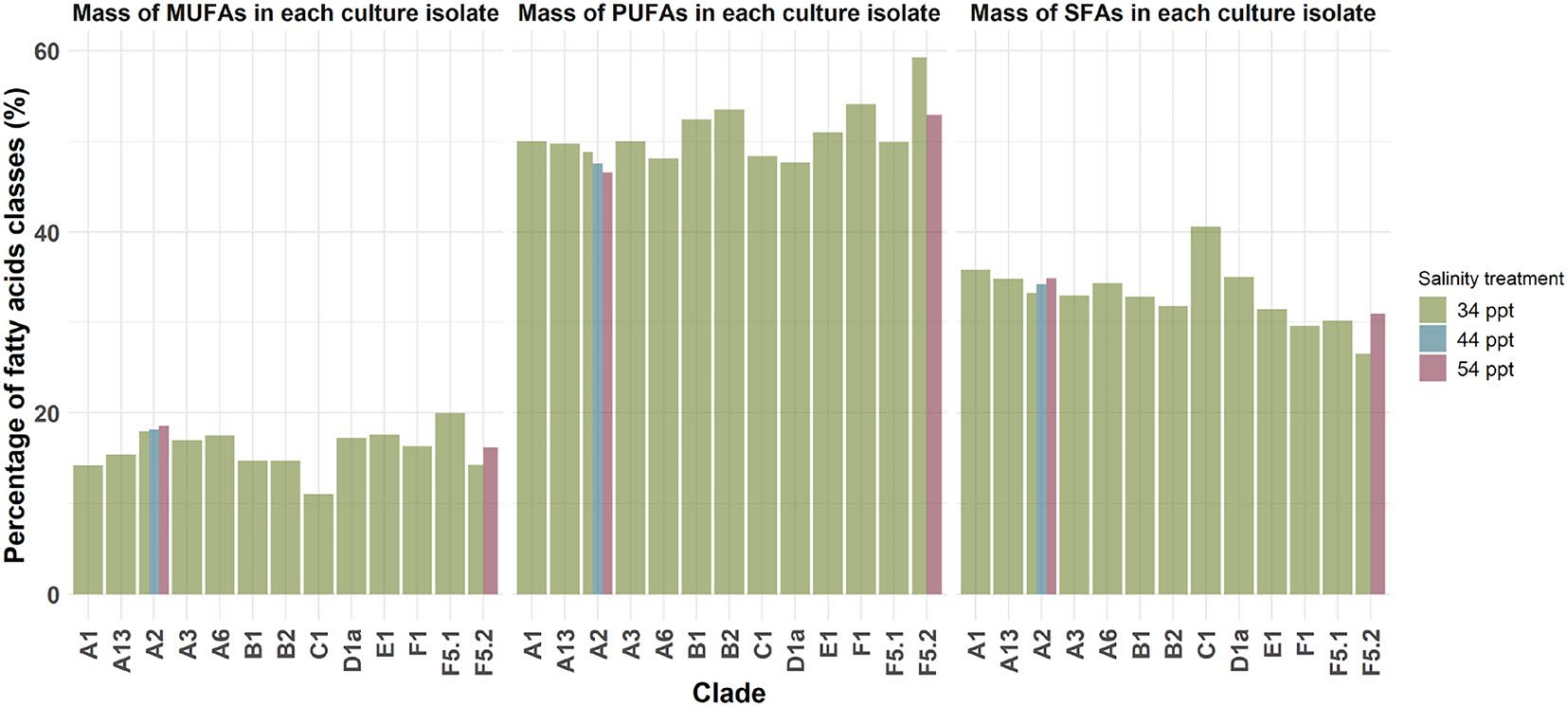
A histogram showing the percentage of the three different fatty acids classes among the culture isolates at 34 ppt (normal salinity) and increased salinities of 44 ppt and 54 ppt for the best performing isolates during cryopreservation. *MUFA* monounsaturated fatty acids, *SFA* saturated fatty acids, *PUFA* polyunsaturated fatty acids, *Ppt* parts per thousand.

Nine different SFAs were identified from C12:0 to C24:0 (Supplementary Information excel Table S1). The mass of the SFAs made up between 26.5 and 40.6% of the total mass of the FAs produced among the culture isolates (Fig. 3). The culture isolate with the highest contribution percentage (14.6%) of SFAs was *D. trenchii* growing at 34 ppt (normal salinity). The highest percentage of all the total SFAs was in *Cladocopium* sp. [C1] (40.6%; Supplementary Information Table S1) and the SFA that was highly expressed was Palmitic acid (C16.0) in *D. trenchii* (Supplementary Information Table S1).

Five different MUFAs were identified from C14:1 to C22:1n9 (Supplementary Information excel Table S1). These MUFAs made up between 11.0 and 19.9% of the total mass of FAs produced among the culture isolates (Fig. 3). The culture isolate with the highest contribution percentage (12.2%) of MUFAs was *D. trenchii* growing at 34ppt (normal salinity). The highest percentage of all the total MUFAs was in *Fugacium* sp. (F5.1; 19.9%) (Supplementary Information Table S1, Fig. 3) and the MUFA that was highly expressed was Cetoleic/erucic acid (C22:1n9) in *D*. *trenchii* (Supplementary Information Table S1).

Ten different PUFAs from C16:2n4 to C22:6n3 were identified. The PUFAs can also be differentiated by omega 3 and omega 6 FAs (Supplementary Information excel Table S1). These PUFAs made up between 45.4 and 59.3% of the total mass of all the FAs produced among the culture isolates (Fig. 3). The culture isolate with the highest contribution percentage (13.0%) of MUFAs was *D. trenchii* growing at 34 ppt (normal salinity). The highest percentage of all the total PUFAs was *Fugacium* sp. at normal (34 ppt) salinity (F5.2; 59.3%) (Supplementary Information Table S1, Fig. 3) and the PUFA that was highly expressed was C18:4n3 stearidonic acid (SDA) in *D. trenchii* (Supplementary Information Table S1).

### Changes in mass of the main classes of fatty acids after high salinity

#### *Symbiodinium pilosum* [A2] in 44 ppt and 54 ppt salinities

**Figure 4:**
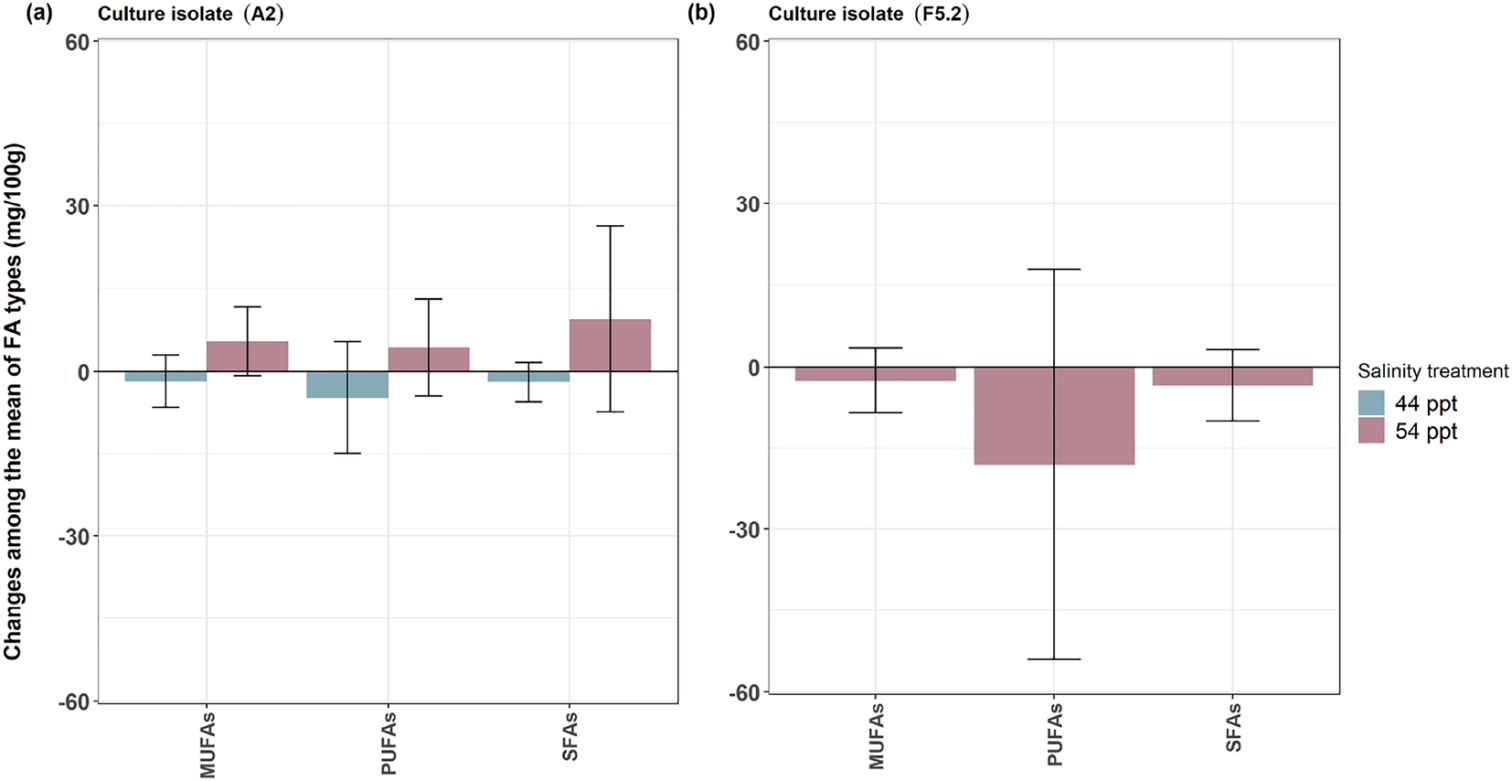
Changes in fatty acid (FA) concentration (mg 100 g^−1^ of FAME production) at different salinity treatments grouped by FA class for all individual FA profiles (MUFA = Monounsaturated fatty acids, SFA = Saturated fatty acids, PUFA = Polyunsaturated fatty acids); (**a**) *Symbiodinium pilosum* [A2] culture isolate after treatment with f/2 media at 44 ppt and 54 ppt salinities in comparison to f/2 media at 34 ppt (normal salinity; zero line). (**b**) *Fugacium* sp. [F5.2] culture isolate after treatment with f/2 media at 54 ppt salinity only in comparison to f/2 media at 34 ppt (normal salinity, zero line).

For the high salinity treatments, our descriptive analysis of the FA analyses after growth in the two increased salinity treatments of 44 ppt and 54 ppt showed major differences in the three main classes of FA when compared to normal salinity. There was higher production of SFAs, MUFAs and PUFAs in 54 ppt salinity compared to normal salinity (Fig. 4a). Conversely, under 44 ppt salinity, these three fatty acid classes were produced in lower amounts compared to normal salinity (Fig. 4a).

#### *Fugacium* sp. [F5.2] in 54 ppt salinity

For the FAs expressed by this culture isolate, descriptively there was a low production of all the three FA classes when the isolate was grown in 54 ppt salinity as compared to normal salinity (Fig. 4b).

### Changes in the mass of individual fatty acids after high salinity

**Figure 5:**
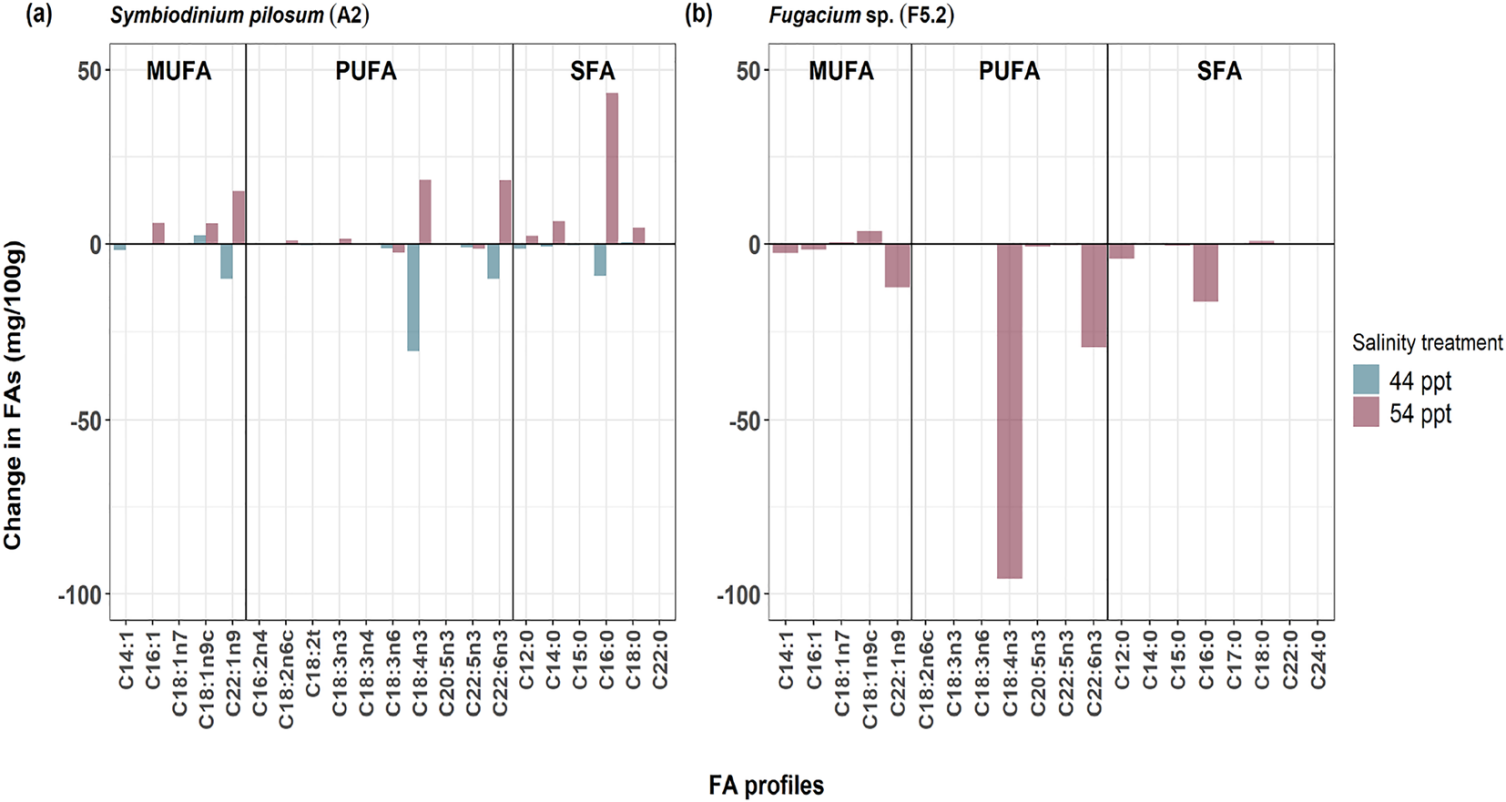
The changes in different fatty acid (FA) profiles of two Symbiodiniaceae culture isolates: (**a**) *Symbiodinium pilosum* [A2] after treatment with f/2 media at 44 ppt and 54 ppt salinities compared to f/2 media at 34 ppt (normal salinity; zero line); (**b**) *Fugacium* sp. [F5.2] after treatment with f/2 media at 54 ppt salinity in comparison to f/2 media at 34 ppt salinity (normal salinity). The values are in mg 100 g^−1^ change of fatty acid methyl esters.

The initial descriptive analyses of the individual FA profiles of the two culture isolates under high salinity treatment showed some changes in the mass of major FA profiles of *S*. *pilosum* [A2] after increased salinity treatments of 44 ppt and 54 ppt as well as *Fugacium* sp. [F5.2] after 54 ppt salinity were compared to those grown in normal salinity. A further experimentation with appropriate replication with strengthen these findings. Changes of less than 2.0 mg 100 g^−1^ between increased and normal salinity were not considered in our comparison because many minor FA detected in our isolates had ranges of 0–2.0 mg 100 g^−1^. Our initial descriptive findings on changes in the FA profiles were visualized in comparative bar graphs (Fig. 5).

#### *Symbiodinium pilosum* [A2] in 44 ppt and 54 ppt salinities

The initial descriptive findings of the FAs profiles of this culture isolate under high salinity treatments, For the major SFAs produced, myristic acid increased by 6.7 mg 100 g^−1^ in 54 ppt salinity compared to normal salinity treatment (Fig. 5a). Palmitic acid (C16:0) decreased by 9.1 mg 100 g^−1^ in 44 ppt salinity but had a large increase of 43.4 mg 100 g^−1^ in 54 ppt salinity compared to normal salinity. Stearic acid (C18:0), increased by 4.6 mg 100 g^−1^ in 54 ppt salinity compared to normal salinity (Fig. 5a). There were changes in production of three MUFAs; palmitoleic acid (C16:1) had an increase of 6.1 mg 100 g^−1^ in 54 ppt salinity treatment as compared to normal salinity (Fig. 5a). The production of oleic acid (C18:1n9c) increased in both salinity treatments by 2.4 mg 100 g^−1^ in 44 ppt and 6.0 mg 100 g^−1^ in 54 ppt salinity treatments respectively (Fig. 5a). Cetoleic/erucic acid (C22:1n9), decreased by 10.0 mg 100 g^−1^ in 44 ppt salinity but in 54 ppt salinity, there was an increase of 15.3 mg 100 g^−1^ compared to normal salinity (Fig. 5a). Higher changes occurred in the mass of some PUFAs, such as stearidonic acid (SDA) (C18:4n3) that decreased sharply by 30.5 mg 100 g^−1^ in 44 ppt salinity treatment, but it increased by 18.4 mg 100 g^−1^ in 54 ppt salinity compared to normal salinity. Docosahexaenoic acid (DHA) (C22:6n3) produced decreased by 10.0 mg 100 g^−1^ in 44 ppt salinity but it increased by 18.3 mg 100 g^−1^ in 54 ppt salinity when these two treatments were compared to normal salinity (Fig. 5a).

#### *Fugacium* sp. (F5.2) in 54 ppt salinity

For this culture isolate, descriptive analyses of the FA profiles showed that in the SFAs, there was a decrease of 4.3 mg 100 g^−1^ in lauric acid (C12:0) in 54 ppt salinity compared to normal salinity treatments. Palmitic acid (C16:0) experienced a decrease of 16.4 mg 100 g^−1^ when this culture was grown in 54 ppt salinity compared to normal salinity (Fig. 5b). Production of the MUFAs; Oleic acid (C18:1n9c), increased by 3.6 mg 100 g^−1^ under this high salinity treatment. In contrast, cetoleic/erucic acid (C22:1n9) production decreased by 12.4 mg 100 g^−1^ in 54 ppt salinity as compared to normal salinity (Fig. 5b). Lastly in PUFAs, stearidonic acid (SDA) (C18:4n3) production decreased sharply by 95.7 mg 100 g^−1^ in 54 ppt salinity treatment as compared to normal salinity. Docosahexaenoic acid (DHA) (C22:6n3) decreased by 29.6 mg 100 g^−1^ in the increased salinity of 54 ppt as compared to normal salinity (Fig. 5b).

## Discussion

The successful cryopreservation of different microalgal species offers an alternative maintenance technique to the conventional serial sub-culturing and is the best method for their long-term storage^47^. In this study, a cryopreservation protocol optimized by Kihika et al. ^10^ using DMSO as the CPA and two freezing techniques, was applied to fifteen culture isolates from the family Symbiodiniaceae. DMSO was the preferred CPA because it can readily pass through the cell membrane into the intracellular space, can be removed easily from the cells, does not cause bacterial contaminations^13,48^ and it has no detrimental effects on the cell health of cryopreserved *Breviolum* sp.^10^. In microalgae, optimum conditions for a successful cryopreservation technique should be examined for each species separately^47^. To successfully cryopreserve the many Symbiodiniaceae cultured isolates with high post thaw survival rates, two main freezing techniques were applied: rapid freezing and controlled-rate freezing.

Before freezing the dinoflagellates, the penetrating CPA, (DMSO) was added in small aliquots to minimize osmotic stress that might affect the cells and their membranes^9,10^. In the first part of this study, all the culture isolates were treated with 15% DMSO before using the rapid freezing and controlled-rate freezer methods. Cultured isolates from *Breviolum*, *Cladocopium, Durusdinium, Effrenium* and *Fugacium* genera were successfully cryopreserved while all the isolates from the genus *Symbiodinium* failed to cryopreserve. All successfully cryopreserved isolates had different cell viabilities after thawing, and this illustrates that the optimum conditions for cryopreservation vary from one species to another^13^.

During cryopreservation, adequate dehydration of the microalgal cells has to be achieved using a suitable concentration of a cryoprotectant for each species^10,13,49^. Lowering of the DMSO concentration from 15 to 10% before freezing led to higher cell viabilities in *Fugacium* sp. [F5.2] in both rapid freezing and the controlled-rate freezer method which suggests that a higher concentration of DMSO might be toxic to this species. Surprisingly, *Symbiodinium microadriaticum* [A1], *S*. *pilosum* [A2] and *S*. *necroappetens* [A13] successfully cryopreserved only in the rapid freezing technique after using a lower concentration of 10% DMSO. These differences in cell survival among the Symbiodiniaceae culture isolates from both 15% and 10% DMSO treatments shows that the best type of CPA and its concentration has to be determined for each species empirically^10^.

High salinity treatments were applied to five *Symbiodinium* culture isolates that failed to cryopreserve with the initial experiments and to *Fugacium* sp. [F5.2] that had very low post thaw viability after using 15% DMSO. These culture isolates were subjected to salinity treatments of 44 ppt, 54 ppt and 64 ppt and then prepared for cryopreservation by being treated with 15% DMSO to facilitate cell dehydration before freezing^10,21^. Both *S. pilosum* [A2] and *Fugacium* sp. [F5.2] cryopreserved with high cell viabilities post thaw after the high salinity treatments. The high survival rates in these two culture isolates after salinity treatments indicates that ions from the dissolved salts may have acted as a dehydrating agent and the treatment with DMSO protects the cells membranes and proteins before freezing^21^. In the rapid freezing method, *S. pilosum* [A2] successfully cryopreserved after both 44 ppt and 54 ppt salinity treatments with very high cell viabilities, while *Fugacium* sp. [F5.2] cryopreserved at both 54 ppt and 64 ppt salinities. The differences in survival rates after cryopreservation in the salinity treated culture isolates show that the effects of salinity will depend on the sensitivity of the marine microalgae to high salt stress^21^. Two cultures *S*. *tridacnidorum* [A3] and *S. tridacnidorum* [A6] did not cryopreserve after any of the increased salinity treatments, which may have been caused by a variety of reasons such as the salinity levels applied were not high enough, the DMSO treatment under high salinity may have been toxic to the cells, or the cells underwent excessive dehydration before freezing.

The best cryopreservation technique for *S. pilosum* [A2] was rapid freezing. Under rapid freezing, the cell viability was very low at normal salinity with 10% DMSO but the viability was very high after increased salinity treatments of both 44 ppt and 54 ppt with 15% DMSO. In *Fugacium* sp. [F5.2], higher cell viabilities occurred in normal salinity treatments with 10% DMSO under both rapid and controlled rate freezing techniques. However, after 54 ppt salinity treatment with 15% DMSO, this culture isolate had a high cell viability after rapid freezing. In high salinity, permeable CPAs like DMSO reduce the osmotic stress in the cells by binding together the solute molecules^21^.

The phylogeny of Symbiodiniaceae has undergone many taxonomic revisions as knowledge on their genetics, morphology and ecology has improved^8,50,51^. The fifteen culture isolates in this study were classified into six out of the eleven known Symbiodiniaceae genera based on the 28S rRNA sequence analysis. The FA profiles of the culture isolates shows an interesting pattern in the genus *Symbiodinium* where five out of seven isolates cluster together while in other genera the isolates are mixed in different clusters. These results suggest that FAMEs analysis could serve as a chemotaxonomic marker for validation of phylogenetic classification in some species^52^. The Symbiodiniaceae culture isolates had higher proportions of PUFAs in both normal and high salinities, The most important FA profiles that were highly expressed and are specific to dinoflagellates were stearidonic acid (SDA) (C18:4n3) and docosahexaenoic acid (DHA) (C22:6n3)^53^. Increased salinity treatment induced *S*. *pilosum* [A2] and *Fugacium* sp. [F5.2] to express FA profiles that were different from those treated with normal salinity. Therefore, increasing salinity modified the type and amount of FA produced. Compared to the FAs produced in normal salinity, both *S. pilosum* [A2] and *Fugacium* sp. [F5.2] culture isolates recorded a sharp decrease in stearidonic acid (SDA) (C18:4n3) at 44 ppt and 54 ppt salinities, which is a glycolipid constituent synthesized within the chloroplast^54^. The reason for this decrease is not clear but we believe it is related to the desaturation of SDA that is an important precursor in the biosynthesis of Eicosapentaenoic acid (EPA) (20:5ω3)^55^. The decreased level of PUFAs in the two culture isolates under those high salinity treatments might have been caused by the cells trying to regulate the lipid phase to prevent disruption of membrane integrity^56^. However, the exact role of fatty acid composition in the osmoregulatory function of microalgal cells is not clear^57^.

There was a large amount of SFAs in *S. pilosum* [A2] after 54 ppt salinity treatment. The increase in SFAs may prevent leakage of compatible solutes out of the cell and diffusion of excess ions into the cell^58^. Alternatively, increasing unsaturated FAs in the membrane may stabilize the photosynthetic machinery of the microalgae under salinity stress conditions^59,60^. The initial investigation of the FA classes showed that the PUFAs were produced in low amounts in both *S. pilosum* [A2] and *Fugacium* sp. [5.2] under 54 ppt salinity. This might have been caused by the cells using these FAs to maintain the fluidity of the cell membrane under high salinity. To confirm these findings and results in the future, an increased number of replicates should be used to conduct appropriate statistical analyses. Overall changes in the sum of the number of double bonds and rings in FAs are important in maintaining the fluidity of the microalgal cell membranes and in providing the appropriate environment for membrane functions^24^. However, the variability in the production of FA classes under increased salinity in the two cultures demonstrates that individual species respond differently to salinity stress levels, and may express their FA classes differently, perhaps as a result of distinct means of acclimation, stress response or changes in the lipid composition of the cells^24^. Many marine microalgal species are able to tolerate great variations of salinity, but their chemical and fatty acid composition varies greatly with respect to increased salt stress^23^.

The changes in the FAs composition of cell membranes can affect their fluidity^24^ and this may therefore have an impact on the ability to cryopreserve different species. The physical properties of fatty acids differ due the length of the carbon chain and the number of double bonds. In general, the longer the FA chain or the more the double bonds, the lower the melting point. Therefore, increases in PUFAs in the FA profiles of these Symbiodiniaceae isolates will theoretically maintain their membrane’s structure^24,25^ and improve their ability to survive during cryopreservation.

## Conclusion

Symbiodiniaceae species are highly diverse, and their existence in natural habitats as well as that of their associated hosts are threatened by the current global climatic crisis. Cryopreservation will help protect these valuable dinoflagellates for future scientific research and establish a seed bank for the conservation of threatened coral reef habitats prioritizing their recovery plans. In this study, we showed that different approaches were required for the Symbiodiniaceae culture isolates including varying the concentrations of DMSO, increasing nutrient medium salinity and application of two different freezing techniques. Our results indicate that, the best cryopreservation technique with the highest survival rates among the Symbiodiniaceae culture isolates was rapid freezing. However, the differential survival rates of some Symbiodiniaceae cultures among the two freezing methods demonstrate that it is unlikely that a common universal cryopreservation protocol for all microalgae can be developed. High salinity treatments of some Symbiodiniaceae isolates show that dehydration of the microalgae cells and the changes in their fatty acid profiles before cryopreservation leads to higher cell viabilities. This may be an important treatment step that should be considered when developing future protocols for Symbiodiniaceae cryopreservation. Application of new approaches to Symbiodiniaceae cryopreservation, such as investigation of their FA profiles and increasing salinity treatments, will greatly improve the current cryopreservation protocols to include more species from other genera and increase their survival rates.

## Supplementary Information

Supplementary Table S1.

Supplementary Figure S1.

## Data Availability

All FA data generated or analysed during this study are included in this published article and its Supplementary Information excel file. All DNA sequences generated in this study are available in the GenBank database (National Center for Biotechnology Information) under the following accession numbers: ITS-2 sequences: ON259675–ON259689. 28S rRNA sequences: ON263271–ON263285.

## References

[1] Coffroth MA, Santos SR (2005). Genetic diversity of symbiotic dinoflagellates in the genus *Symbiodinium*. Protist.

[2] Pochon X (2010). Comparison of endosymbiotic and free-living *Symbiodinium* (Dinophyceae) diversity in a Hawaiian reef environment^1^. J. Phycol..

[3] Kemp DW (2015). Spatially distinct and regionally endemic *Symbiodinium* assemblages in the threatened Caribbean reef-building coral *Orbicella faveolata*. Coral Reefs.

[4] Mansfield KM, Gilmore TD (2019). Innate immunity and cnidarian-Symbiodiniaceae mutualism. Dev. Comp. Immunol..

[5] Pochon X, Gates RD (2010). A new *Symbiodinium* clade (Dinophyceae) from soritid foraminifera in Hawai’i. Mol. Phylogenet. Evol..

[6] Yorifuji M (2021). Unique environmental Symbiodiniaceae diversity at an isolated island in the northwestern Pacific. Mol. Phylogenet. Evol..

[7] Qin Z (2019). Diversity of Symbiodiniaceae in 15 coral species from the southern south China sea: Potential relationship with coral thermal adaptability. Front. Microbiol..

[8] Pochon X, LaJeunesse TC (2021). Miliolidium n. gen, a new Symbiodiniacean genus whose members associate with soritid foraminifera or are free-living. J. Eukaryotic Microbiol..

[9] Rhodes L (2006). Cryopreservation of economically valuable marine micro-algae in the classes Bacillariophyceae, Chlorophyceae, Cyanophyceae, Dinophyceae, Haptophyceae, Prasinophyceae, and Rhodophyceae. Cryobiology.

[10] Kihika JK (2022). Cryoprotectant treatment tests on three morphologically diverse marine dinoflagellates and the cryopreservation of *Breviolum* sp. (Symbiodiniaceae). Sci. Rep..

[11] Tsai S, Lin C (2012). Advantages and applications of cryopreservation in fisheries science. Braz. Arch. Biol. Technol..

[12] Abreu L, Borges L, Marangoni J, Abreu PC (2012). Cryopreservation of some useful microalgae species for biotechnological exploitation. J. Appl. Phycol..

[13] Taylor R, Fletcher RL (1999). Cryopreservation of eukaryotic algae—A review of methodologies. J. Appl. Phycol..

[14] Santiago-Vázquez LZ, Newberger NC, Kerr RG (2007). Cryopreservation of the dinoflagellate symbiont of the octocoral *Pseudopterogorgia elisabethae*. Mar. Biol..

[15] Chong G, Tsai S, Wang L-H, Huang C-Y, Lin C (2016). Cryopreservation of the gorgonian endosymbiont *Symbiodinium*. Sci. Rep..

[16] Cirino L (2019). First instance of settlement by cryopreserved coral larvae in symbiotic association with dinoflagellates. Sci. Rep..

[17] Thongpoo P, Tsai S, Lin C (2019). Assessing the impacts of cryopreservation on the mitochondria of a thermotolerant *Symbiodinium* lineage: Implications for reef coral conservation. Cryobiology.

[18] Lin C (2019). Cryopreservation of a thermotolerant lineage of the coral reef dinoflagellate *Symbiodinium*. Biopreserv. Biobanking.

[19] Di Genio S, Wang L-H, Meng P-J, Tsai S, Lin C (2021). “Symbio-Cryobank”: Toward the development of a cryogenic archive for the coral reef dinoflagellate symbiont Symbiodiniaceae. Biopreserv. Biobanking.

[20] Li H-H, Lu J-L, Lo H-E, Tsai S, Lin C (2021). Effect of cryopreservation on proteins from the ubiquitous marine dinoflagellate *Breviolum* sp. (Family Symbiodiniaceae). Plants.

[21] Cañavate JP, Lubian LM (1995). Relationship between cooling rates, cryoprotectant concentrations and salinities in the cryopreservation of marine microalgae. Mar. Biol..

[22] BenMoussa-Dahmen I, Chtourou H, Rezgui F, Sayadi S, Dhouib A (2016). Salinity stress increases lipid, secondary metabolites and enzyme activity in *Amphora subtropica* and *Dunaliella* sp. for biodiesel production. Bioresource Technol..

[23] Mohan SV, Devi MP (2014). Salinity stress induced lipid synthesis to harness biodiesel during dual mode cultivation of mixotrophic microalgae. Biores. Technol..

[24] Xu X-Q, Beardall J (1997). Effect of salinity on fatty acid composition of a green microalga from an antarctic hypersaline lake. Phytochemistry.

[25] Zhang P (2011). A novel omega-3 fatty acid desaturase involved in acclimation processes of polar condition from antarctic ice algae *Chlamydomonas* sp. ICE-L. Mar. Biotechnol..

[26] Morris GJ (1981). Cryopreservation: An Introduction to Cryopreservation in Culture Collections.

[27] Yamada T (2002). Roles of the plasma membrane and the cell wall in the responses of plant cells to freezing. Planta.

[28] Cronan JE, Gelmann EP (1975). Physical properties of membrane lipids: biological relevance and regulation. Bacteriol. Rev..

[29] Ishika T, Moheimani NR, Laird DW, Bahri PA (2019). Stepwise culture approach optimizes the biomass productivity of microalgae cultivated using an incremental salinity increase strategy. Biomass Bioenerg..

[30] Rhodes L (2016). The Cawthron Institute Culture Collection of micro-algae: A significant national collection. NZ J. Mar. Freshwat. Res..

[31] Guillard, R. R. L. Culture of Phytoplankton for Feeding Marine Invertebrates. in *Culture of Marine Invertebrate Animals* (eds M.L. Smith & M.H. Chanley). (Springer, Boston, MA. 1975). 10.1007/978-1-4615-8714-9_3

[32] Guillard RRL, Hargraves PE (1993). *Stichochrysis immobilis* is a diatom, not a chrysophyte. Phycologia.

[33] van der Merwe R (2014). High salinity tolerance of the Red Sea coral *Fungia granulosa* under desalination concentrate discharge conditions: an in situ photophysiology experiment. Front. Mar. Sci..

[34] Pochon X, Wecker P, Stat M, Berteaux-Lecellier V, Lecellier G (2019). Towards an in-depth characterization of Symbiodiniaceae in tropical giant clams via metabarcoding of pooled multi-gene amplicons. PeerJ.

[35] Pochon X, Pawlowski J, Zaninetti L, Rowan R (2001). High genetic diversity and relative specificity among *Symbiodinium*-like endosymbiotic dinoflagellates in soritid foraminiferans. Mar. Biol..

[36] Pochon X, Garcia-Cuetos L, Baker AC, Castella E, Pawlowski J (2007). One-year survey of a single Micronesian reef reveals extraordinarily rich diversity of *Symbiodinium* types in soritid foraminifera. Coral Reefs.

[37] Stat M, Pochon X, Cowie R, Gates R (2009). Specificity in communities of *Symbiodinium* in corals from Johnston Atoll. Mar. Ecol. Prog. Ser..

[38] Pochon X, Putnam HM, Gates RD (2014). Multi-gene analysis of *Symbiodinium* dinoflagellates: A perspective on rarity, symbiosis, and evolution. PeerJ.

[39] Hall T (1999). BioEdit: A user-friendly biological sequence alignment editor and analysis program for Windows 95/98/NT. Nucleic Acids Symp..

[40] Kumar S, Stecher G, Li M, Knyaz C, Tamura K (2018). MEGA X: Molecular evolutionary genetics analysis across computing platforms. Mol. Biol. Evol..

[41] Felsenstein J (1985). Confidence limits on phylogenies: An approach using the bootstrap. Evolution.

[42] Parrish CC, Nichols PD, Pethybridge H, Young JW (2015). Direct determination of fatty acids in fish tissues: Quantifying top predator trophic connections. Oecologia.

[43] Miller MR (2020). Bioavailability of orally administered active lipid compounds from four different greenshell™ mussel formats. Mar. Drugs.

[44] Masood A, Stark KD, Salem JN (2005). A simplified and efficient method for the analysis of fatty acid methyl esters suitable for large clinical studies. J. Lipid Res..

[45] Ketchen DJ, Shook CL (1996). The application of cluster analysis in strategic management research: An analysis and critique. Strateg. Manag. J..

[46] Tibshirani R, Walther G, Hastie T (2001). Estimating the number of clusters in a data set via the gap statistic. J. R. Stat. Soc. Series B Stat. Methodol..

[47] Youn J-Y, Hur S-B (2009). Cryopreserved marine microalgae grown using different freezing methods. Algae.

[48] Stock W (2018). Expanding the toolbox for cryopreservation of marine and freshwater diatoms. Sci. Rep..

[49] Nakanishi K, Deuchi K, Kuwano K (2012). Cryopreservation of four valuable strains of microalgae, including viability and characteristics during 15 years of cryostorage. J. Appl. Phycol..

[50] LaJeunesse, T. C. *et al.* Revival of *Philozoon* Geddes for host-specialized dinoflagellates, ‘zooxanthellae’, in animals from coastal temperate zones of northern and southern hemispheres. *Eur. J. Phycol*. 1–15 (2021).

[51] LaJeunesse TC (2018). Systematic revision of Symbiodiniaceae highlights the antiquity and diversity of coral endosymbionts. Curr. Biol..

[52] Sharma B, Arora S, Sahoo D, Deswal R (2020). Comparative fatty acid profiling of Indian seabuckthorn showed altitudinal gradient dependent species-specific variations. Physiol. Mol. Biol. Plants.

[53] Papina M, Meziane T, van Woesik R (2003). Symbiotic zooxanthellae provide the host-coral *Montipora digitata* with polyunsaturated fatty acids. Comp. Biochem. Physiol. B Biochem. Mol. Biol..

[54] Leblond JD, Chapman PJ (2000). Lipid class distribution of highly unsaturated long chain fatty acids in marine dinoflagellates. J. Phycol..

[55] Jónasdóttir SH (2019). Fatty acid profiles and production in marine phytoplankton. Mar. Drugs.

[56] Lee Y-K, Tan H-M, Low C-S (1989). Effect of salinity of medium on cellular fatty acid composition of marine alga *Porphyridium cruentum* (Rhodophyceae). J. Appl. Phycol..

[57] Renaud SM, Parry DL (1994). Microalgae for use in tropical aquaculture. II: Effect of salinity on growth, gross chemical composition and fatty acid composition of three species of marine microalgae. J. Appl. Phycol..

[58] Elenkov I, Stefanov K, Dimitrova-Konaklieva S, Popov S (1996). Effect of salinity on lipid composition of *Cladophora vagabunda*. Phytochemistry.

[59] Allakhverdiev SI, Kinoshita M, Inaba M, Suzuki I, Murata N (2001). Unsaturated fatty acids in membrane lipids protect the photosynthetic machinery against salt-induced damage in *Synechococcus*^1^. Plant Physiol..

[60] Barati B, Gan SY, Lim PE, Beardall J, Phang SM (2019). Green algal molecular responses to temperature stress. Acta Physiol. Plant..

