# Supplementary material for: Cryopreservation of six Symbiodiniaceae genera and assessment of fatty acid profiles in response to increased salinity treatments
Source: Sci Rep. 2022 Jul 20;12:12408. doi: 10.1038/s41598-022-16735-w (PMC9300622; doi:10.1038/s41598-022-16735-w)
Supplement: Supplementary file 2 — Supplementary Figure S1. [file 41598_2022_16735_MOESM2_ESM.docx]

**Supplementary Information**

**Cryopreservation of six Symbiodiniaceae genera and assessment of fatty acid profiles in response to increased salinity treatments**

**Joseph Kanyi Kihika^*,1,2^, Susanna A. Wood^1^, Lesley Rhodes^1^, Kirsty F. Smith^1,3^, Matthew R. Miller^1^, Xavier Pochon^1,4^, Lucy Thompson^1^, Juliette Butler^1^, Jessica Schattschneider^1^, Clint Oakley^2^ and Ken G. Ryan^2^**

^1^ Cawthron Institute, Private Bag 2, Nelson 7042, New Zealand

^2^ School of Biological Sciences, Victoria University of Wellington, PO Box 600, Wellington 6140, New Zealand

^3^ School of Biological Sciences, University of Auckland, Private Bag 92019, Auckland 1142, New Zealand

^4^ Institute of Marine Science, University of Auckland, Private Bag 349, Warkworth 0941, New Zealand

***** Correspondence: joseph.kihika@vuw.ac.nz

**Selection of the *k*-means for the fatty acid clusters in Symbiodiniaceae culture isolates**


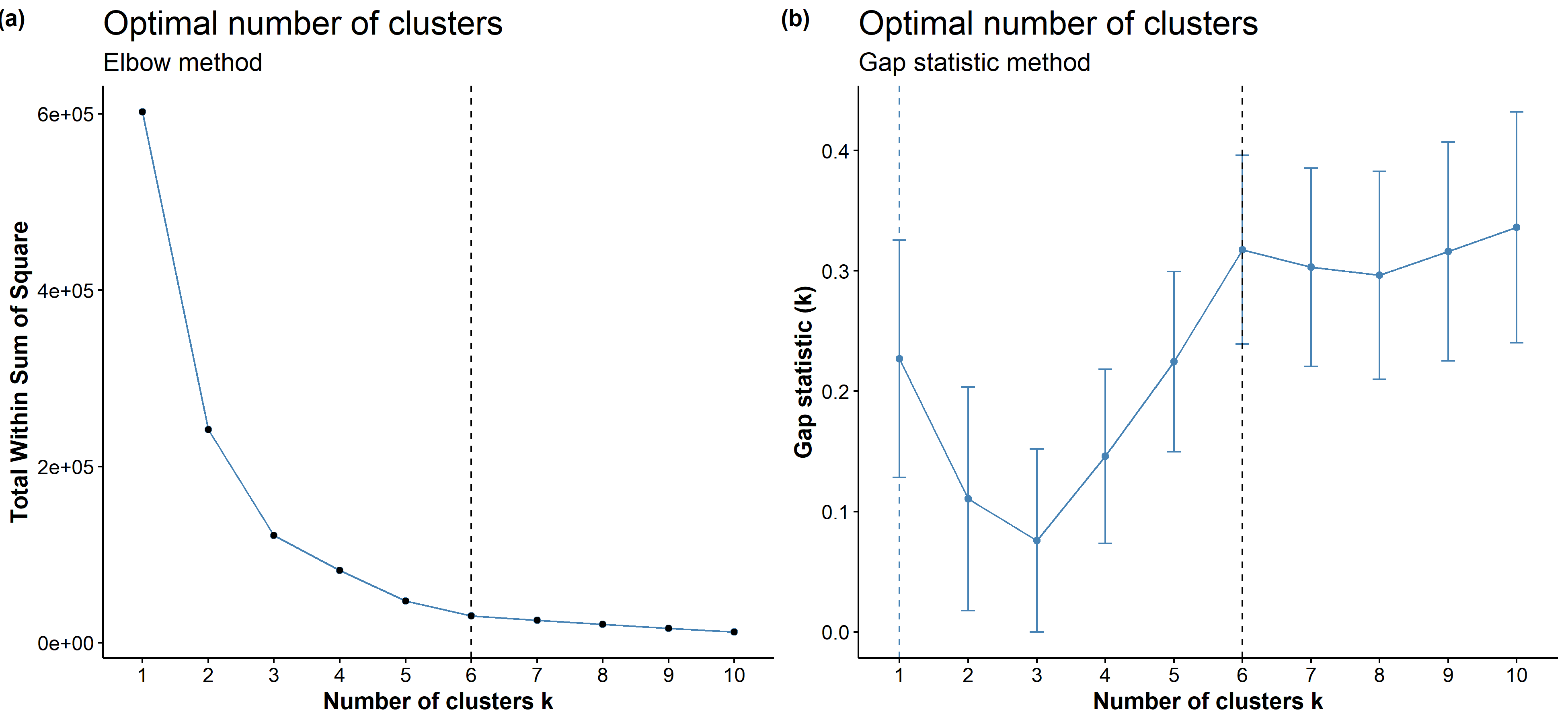


**Figure S1**. Graphs showing the results of the *k*-means when selecting the number of clusters for the descriptive analysis of the FAs profiles produced by the Symbiodiniaceae culture isolates at normal and high salinity treatments before cryopreservation. *k*-means = 6.
